# Overlengthening of the radial column in radial head replacement: a review of the literature and presentation of a classification system

**DOI:** 10.1007/s00402-020-03619-9

**Published:** 2020-10-14

**Authors:** K. Wegmann, M. Hackl, T. Leschinger, K. J. Burkhart, L. P. Müller

**Affiliations:** 1 Faculty of Medicine and University Hospital, Center for Orthopedic and Trauma Surgery, University Medical Center of Cologne, Kerpener Street 62, 50937 Cologne, Germany; 2 Arcus Sportklinik, Pforzheim, Germany

**Keywords:** Overlengthening, Radial head, Arthroplasty, Elbow, Erosion

## Abstract

**Background:**

Radial head arthroplasty is a common procedure in elbow surgery. It has been shown to be of benefit for the patients, but there also are relevant complications that should be prevented if possible. One significant complication is overlengthening of the radial head prosthesis. In overlengthening, the head of the prosthesis overextends the physiological level of the native radial head and leads to overcompression in the radiohumeral joint. Rapid erosion and arthritic changes may then impede the clinical outcome. The incidence of overlengthening is not precisely known, but estimations range to up to 20% of all implanted prostheses.

**Methods:**

The present review discusses the available body of literature on overlengthening and lines out a classification system that may be used to guide treatment algorithms. The classification is based on the personal experiences of the author during their clinical practice.

**Results:**

In low-grade overlengthening (type I) conservative treatment can be an option. In Types II–IV usually revision surgery is needed. Depending on the state of the capitulum and joint stability, it is possible re-implant a prosthesis, or rely on implant removal alone.

**Discussion:**

The present review aimed at shedding light into overlengthening as a complication radial head replacement and to help identify and treat it.

## Introduction

Prosthetic implants are commonly in use for the treatment of pathologies of the proximal radius and have been investigated in several clinical studies [1–4]. The safety of the devices and the positive effects on pain, joint stability and load sharing across the joint and the forearm have been well documented. Despite the good impact the introduction and ongoing engineering of the implants had on patient treatment, specific complications related to radial head replacement are well known. Besides shaft-loosening and even uncoupling of prosthesis components, erosion of the capitulum is a common downside when the radial head is replaced. Erosion of the capitulum may lead to pain and stiffness with impaired functionality of the joint. One reason for the erosion of the capitulum, undoubtedly, is the difference in elasticity modules of the native capitulum and the prosthetic material. The amount of erosion can be greatly increased, however, when the pressure between prosthesis and capitulum exceeds physiological limits. This typically is the case in overlengthening of the proximal radius by the implants. Overlengthening occurs either by imperfect implantation, or if longitudinal stability of the forearm is disturbed. Needless to say, the latter is less frequently present, like in Essex-Lopresti lesions. In the case of increased pressure between the radial column and a radial head prosthesis due to longitudinal instability of the forearm, we use the term dynamic overlengthening. Even if a prosthesis of the radial head is implanted correctly, due to radioulnar longitudinal shifting, the prosthesis will abut against the capitulum and erode it. The second reason for overlengthening as mentioned is imperfect implantation. Either there is insufficient resection of bone of the proximal radius before implanting the device or the length of the implant is chosen as such, that the final height of the construct exceeds the height of the resected bone or the traumatic bony defect. Both scenarios lead to an increase in pressure within in the radio-humeral joint resulting in rapid erosion of the capitulum. This condition usually leads to severely impaired function of the joint [5–9]. The clinical study of Schnetzke et al. also showed that overlengthening comes along with impaired clinical outcome [10].

Aside from the term “overlengthening”, this pathology is frequently referred to as “overstuffing” in the available literature. But while overlengthening clearly describes the biomechanical problem at the radio-humeral joint, overstuffing may also be present if the diameter of an implant is too large but sitting at the correct height. Thus, we prefer the term overlengthening, as it more clearly separates the two entities.

As overlengthening is not well investigated and its role in the outcome of radial head prosthetic replacement has not been finally clarified, the present review aims to shed light into its pathomechanics, diagnostics and therapeutic options. In addition, a classification system is presented that shall help to stratify the entity and guide its treatment.

### Epidemiology

The precise incidence of overlengthening is unknown, as it has not been investigated in larger clinical series. Still, the present literature has recognized it as a detrimental complication of radial head arthroplasty. Besides loosening and component dissociation, van Riet et al. found 11 cases with signs of overlengthening in their case series of 47 radial head prostheses, making up for 23% [8]. The group stated that clinical causes of failure like stiffness and pain might well be associated with mal-implantation like overlengthening. In another retrospective report, Hackl et al. investigated the cases of 466 patients, who had suffered fractures of the radial head and had to undergo revision surgery for the initial treatment [11]. In a subgroup of 76 patients who had been initially treated with prosthetic radial head replacement, the authors found radiological evidence of overlengthening in 16 patients, making up for 21%. In a systematic review on the general topic of radial head prosthetic replacement, Heijink et al. included 30 studies reporting on 727 patients and reported that in 56 patients revision was necessary, with again 20% being revised for overlengthening. Kachooei et al. reviewed 30 studies including an overall of 1017 patients who had a radial head prosthesis implanted [12]. Out of these patients in 122 the implant was removed or revised, with overlengthening being the cause in 13% of the cases. At hand of these studies, a precise estimation of the incidence of overlengthening of radial head prostheses is not reliably possible. But the reported numbers show that today, overlengthening most likely is not a rare condition, being at least responsible for 13–23% of radial revisions. And likely it is an underreported condition, as asymptomatic or undiagnosed patients may be present.

### Pathobiomechanics

**Fig. 1:**
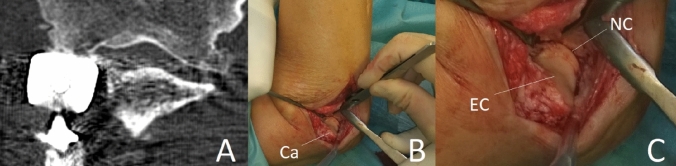
**a** CT-Scan of overlengthened radial head implant, overreaching the lateral edge of the coronoid—with erosion of the capitulum. **b** Intra-operative (Ca: Capitulum). **c** Magnification of the intra-operative findings. *EC* eroded cartilage, *NC* native cartilage of the trochlea

The radial head is not only a significant valgus stabilizer, but also important for longitudinal force transmission, from the wrist through the forearm into the distal humerus. In the healthy distal forearm, ~ 70% of the weight picked up by the wrist is transferred into the distal radius [13]. While the forces travel in a proximal direction through the forearm, the interosseus membrane aids in shifting the load from the radius to the ulna. By that shift, the load distribution at the level of the elbow joint is almost equal for the radial and the ulnar joint compartment [13, 14]. This biomechanical relation is based on the complex anatomy of the joint, where the greater sigmoid notch of the ulna and the radial head articulate intimately with their respective counterparts of the distal humerus, the trochlea and the capitulum. In the native joint, the articular surface of the radial head sits at the level of the lateral edge of the coronoid process, allowing it to have medium contact pressures of 1 to about 1.75 mPa, according to the in vitro study of Cohn et al. [15]. Yet, any changes to that balance result in significant increases in the pressure distribution. In their study, Cohn et al. put the specimens under a load of 4.5 kg, each via biceps/brachialis- and triceps-loading [15]. Overlengthening of the radial column of more than 2 mm led to a significant increase of the contact pressure between the radial head and the capitulum, according to the authors. These in-vitro results comprehensibly explain the clinically observed changes to the capitulum in a joint with an overlengthened prosthesis, where the implant grinds its way into the capitulum (Fig. 1). However, also the contact mechanics between the cartilage and the radial head implant is of relevance. Implantation of a radial head prosthesis means hemi-prosthetic replacement of the joint. Hence, the mechanical properties of the two joint components differ significantly, in terms of their elasticity modulus. While Cobalt/Chrome (e.g., CRF II, Tornier) has a young’s modulus of 210 GPa and Pyrocarbon (e.g., Mopyc, Tornier) of about 25 GPa, articular cartilage has a young’s modulus of 0.5–0.9 MPa and is with that several thousand times lower. By that, erosion of the capitulum is likely to happen anyhow, as a harder material will always grind into the softer material. The study of Burkhart et al. showed a high number of capitellar erosions at an average of 8 years after radial head replacement, even in absence of overlengthening [16]. We know from several biomechanical studies that, besides the differing young’s modulus, compared to the physiological state the contact area between the native capitulum and the radial side is significantly reduced, when commonly available radial head prostheses are implanted [17–21]. As a logical consequence, reduced contact area and a discrepancy in young’s modulus can well lead to abrasion, with its clinical consequences. However, if these two factors are combined with increased load, as is the fact in overlengthening, peak contact pressure per area will increase drastically.

### Diagnostics and classification

#### Clinical signs

Patients may present in varying intervals after radial head replacement and report pain or discomfort, concentrated over the lateral side of the elbow. In severe overlengthening, with closure of the medial joint space, even medial sided pain may be present. Also, crepitus is often noticed. The investigator can reproduce crepitus by rotating the forearm, therewith exerting rotational stress with the prosthesis on the capitulum. This may also lead to provocation of pain; hence the manoeuvre should be performed carefully and with respect to the patient’s symptoms. Both, crepitus and pain can be increased by contraction of the forearm-muscles, e.g. by firm fist-closure. This muscle contraction leads to an increase of the compressive force between capitulum and prosthesis with likely enhancement of the symptoms.

The patients often also present with restricted range of motion in the elbow. Extension/flexion and pronation/supination may be limited in varying degrees. Several factors contribute to the problem. First, as overlengthening often results in erosion of the capitulum, it contributes to joint inflammation. Inflammatory processes usually already are present because of the surgical intervention with implantation of the prosthesis. The inflammation often ends up in scarring of the joint and therewith stiffness. As a second effect of overlengthening, the increased pressure at the capitulum is able to limit rotation by the mechanical effect of increased friction. Same is possible for a flexion deficit, when the overlengthened implant abuts at the ventral aspect of the capitulum in flexion, leading to a mechanical block. This entity was already observed and reported by Birkedahl et al., who stated that the loss of the radio-capitellar joint gap leads to a closure and an abutment in flexion [5]. As a third effect, the pain itself may prevent patients from moving adequately and from proper rehab, by that resulting in stiffness, too.

But the clinical effects of overlengthening are not solely confined to the elbow itself. By the direct biomechanical cohesions between the elbow and the wrist, it is reasonable that overlengthening of the radial column affects the distal forearm with potential negative clinical consequences. With significant radial lengthening the patients might have pain due to the altered radio-ulnar variance. Clinically this may lead to tensioning pain throughout the forearm and constant pain over the DRUJ. Also shooting pain over the DRUJ or wrist is reported by the patients.

#### Imaging

**Fig. 2:**
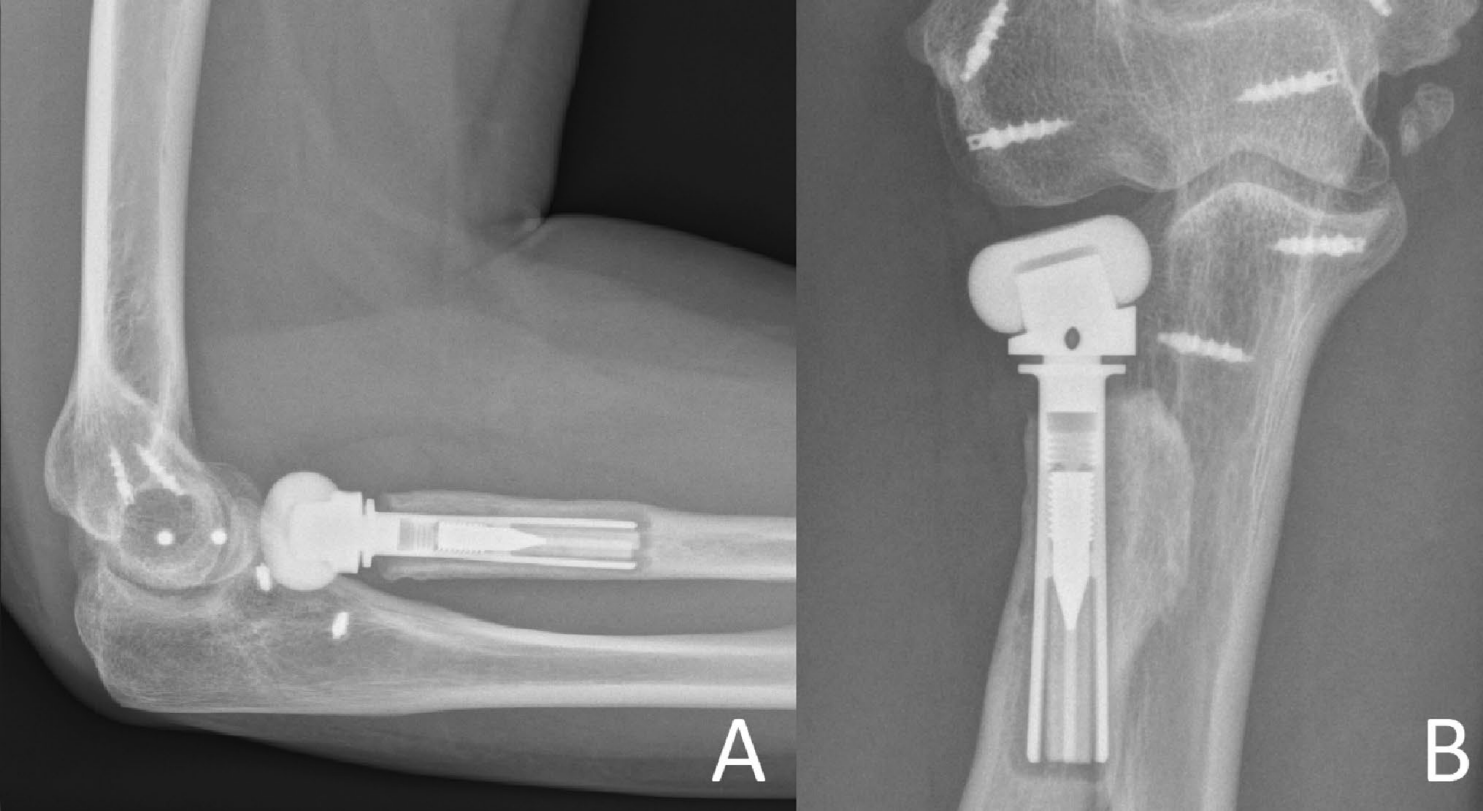
X-ray of substantial loosening of a monopolar radial head prosthesis at the shaft of the proximal radius (**a** lateral, **b** a.p)

The basis of imaging is plain x-rays. The elbow must be filmed in two orthogonal planes, a.p. and strictly lateral. Lateral images are best achieved with the patient sitting and the elbow placed on the table, when the shoulder is abducted at exactly 90°. Therefore, the X-ray table or film must be lifted, but then strictly lateral images are achieved in a reliable fashion. On lateral images, besides overlengthening, joint stability can be evaluated. Joint subluxation can be detected by the so-called “drop-sign” (Fig. 8, black arrow) in the humeroulnar joint cavity. It points towards postero-lateral instability of the ulno-humeral joint, with the displacement of the radial head past the diameter of the capitellum in lateral radiographs (Fig. 8, white circle/line). Also, the ulna “drops” away from the trochlea of the humerus, indicating ulno-humeral instability. When looking for overlengthening, the X-rays should initially be investigated regarding the radial head prosthesis itself. It is of relevance if the implant is firmly fixed or if there are signs of loosening (Fig. 2). An important sign of loosening is osteolysis around the implant and cortical thinning at the proximal radius, which can happen by chronic movement of the shaft of the prosthesis in the radius. Fractures of the shaft by bone loss due to loosening may be present but may also be subtle and should not be overlooked. Also, the remaining length of the bonestock of the proximal radius is of interest for revision surgery.

Resorption must be evaluated, or bone loss due to previous surgeries. Bone loss can be identified by using the biceps tuberosity as landmark. By evaluating the remaining bone proximal to the tuberosity, one can estimate an amount of bone loss. Further, one should look for already implanted bone anchors, whose removal might lead to a loss of bone substance at the distal humerus and would therewith lead to problems during revision surgery when augmenting the ligamentous stabilizers. Such information is of great relevance when planning potential revision surgery.

**Fig. 3:**
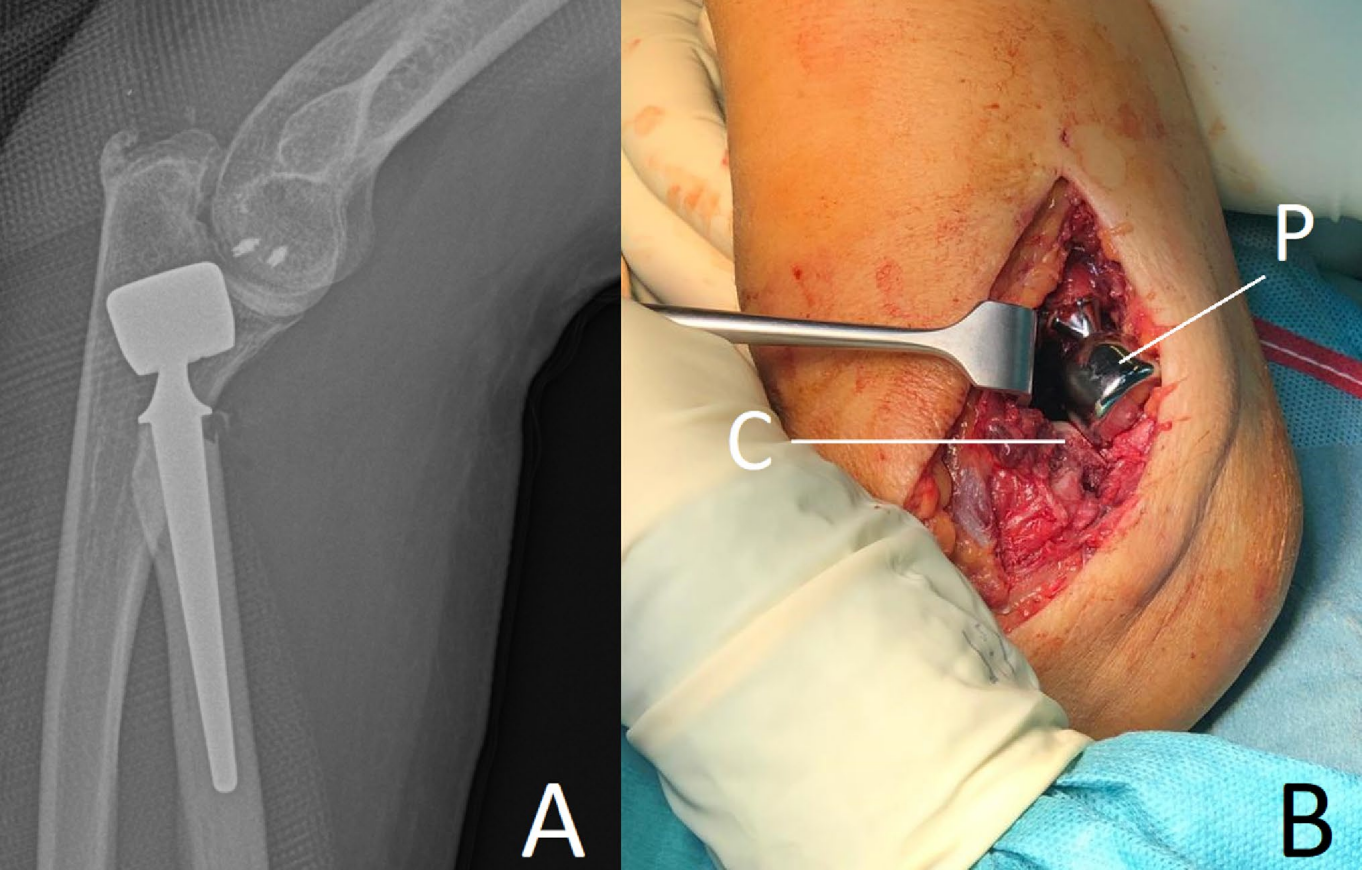
Example of high-grade overlengthening, where the immense pressure between the prosthesis and the capitulum leads to the implant being pushed out dorsally. **a** Lateral X-ray. **b** Intraoperative findings. *C* capitulum, *P* prosthetic implant

Further, there are direct signs of overlengthening to be drawn from the plain X-rays. The investigator should look for signs of proper articulation of the prosthesis with its bony counterparts, as the radial head implant should be aligned centrally under the capitulum in both the a.p. and the lateral plain. In severe overlengthening, the radial head implant may even be pushed out the posterior aspect of the joint (Fig. 3).

**Fig. 4:**
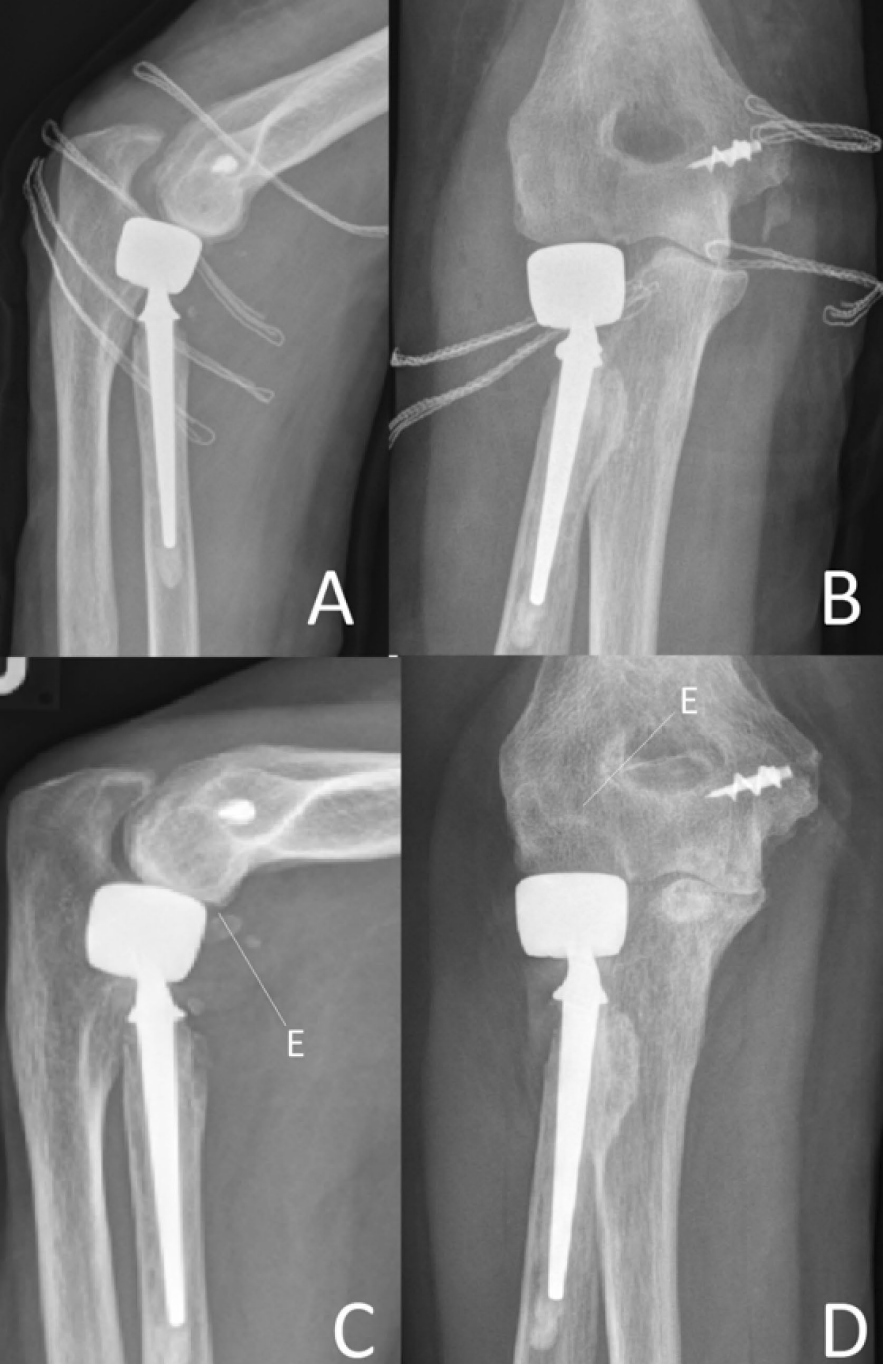
**a**, **b** Show post-OP X-rays after bi-polar radial head replacement. Significant overlengthening is visible. It is shown by the head component of the prosthesis exceeding the level of the lateral edge of the coronoid. Also, subluxation is present. The bony structure of the capitulum is intact, but there is no cartilage cover visible anymore. **c**, **d** show the same patient 1 year later, where the overlengthening has led to abrasion of the cartilage and some of the subchondral bone. In the lateral image (**c**), one can see how a spur has developed (**e**) at the ventral aspect of the capitulum. In the a.p. (**d**) the abrasion has a half-circle shaped form (**e**), resulting from flexion motion of the prosthetic head on the capitulum. To this half-circle shaped X-ray finding at the capitulum in the a.p.-view we refer as the “crescent sign”

The images also help in identifying a loss of parallelism of the ulno-humeral joint line. This loss of parallelism, with a widening in the lateral aspect of the ulno-humeral joint and a closure of the medial aspect, is indicative of an overlengthening of the radial column. The work of Frank et al. showed in a cadaver study that parallelism of the ulno-humeral joint line was lost in overlengthening of the radial column [22]. This most likely results from the overlengthened prosthesis pushing against the capitulum, unhinging the ulno-humeral joint. However, the authors found that a visible difference in the parallelism of the ulno-humeral joint line in X-rays was only present in overlengthening of 6 mm or more. Further studies have taken on the understanding of overlengthening in imaging and how to identify it reliably. Athwal et al. published a method using X-ray imaging of the contra-lateral elbow to compare the ulno-humeral alignment in the a.p.-view to check for overlengthening in mono-axial radial head prostheses [23]. With their method, according to the authors, it is possible to quantify the magnitude of overlengthening; in this paper the authors used the Evolve prosthesis (Wright Medical). Another group reported on the usefulness of the ulnar variance, to detect overlengthening [24]. Moon et al. could show that increasing radial length by overlengthening of a radial head implant leads to a decrease of ulnar variance. So, if in question, X-ray imaging of both wrists may add some information. However, in our own study on the influence of overlengthening on ulnar variance and loss of parallelism, we could not replicate these results [25]. Several other authors have done anatomical studies to define anatomical landmarks that can be used on X-ray to identify or quantify overlengthening [26, 27]. Doornberg et al. investigated native CT-scans of 17 elbows to analys**e the anatomic relation of the radial head to the lateral edge of the coronoid [26]. They found that the radial head is levelled in average at 0.9 mm above the lateral edge of the coronoid. The authors recommend placing a prosthetic implant at the level of, or only slightly above the lateral edge of the coronoid to avoid overlengthening of the radial column. Another sign of overlengthening is abrasion of the cartilage, the subchondral bone or even the bone stock of the capitulum, as a result of the severely increased contact pressure. Even if not present initially, the abrasion of the capitulum may take place over time (Fig. 4) and may therewith limit surgical options for revision in the future.

### Classification

We propose the following classification system, to be able to differentiate between minor to extensive overlengthening and to take chronic changes of the capitulum into account.

**Fig. 5:**
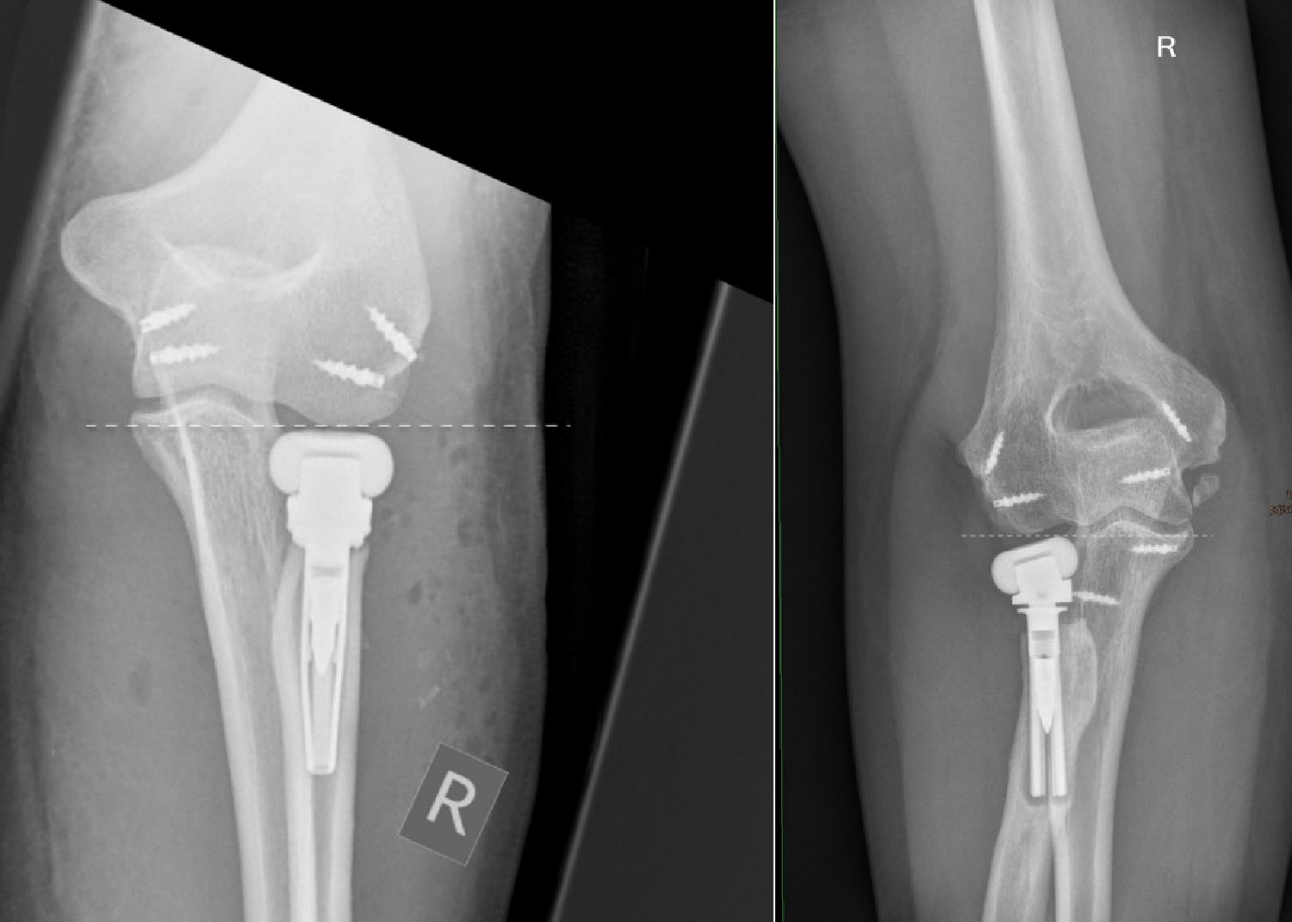
Measurements of the anatomic relation of the radial head to the lateral edge of the coronoid (white dotted line). In these cases the radial prosthesis is slightly beneath the lateral edge of the coronoid

**Fig. 6:**
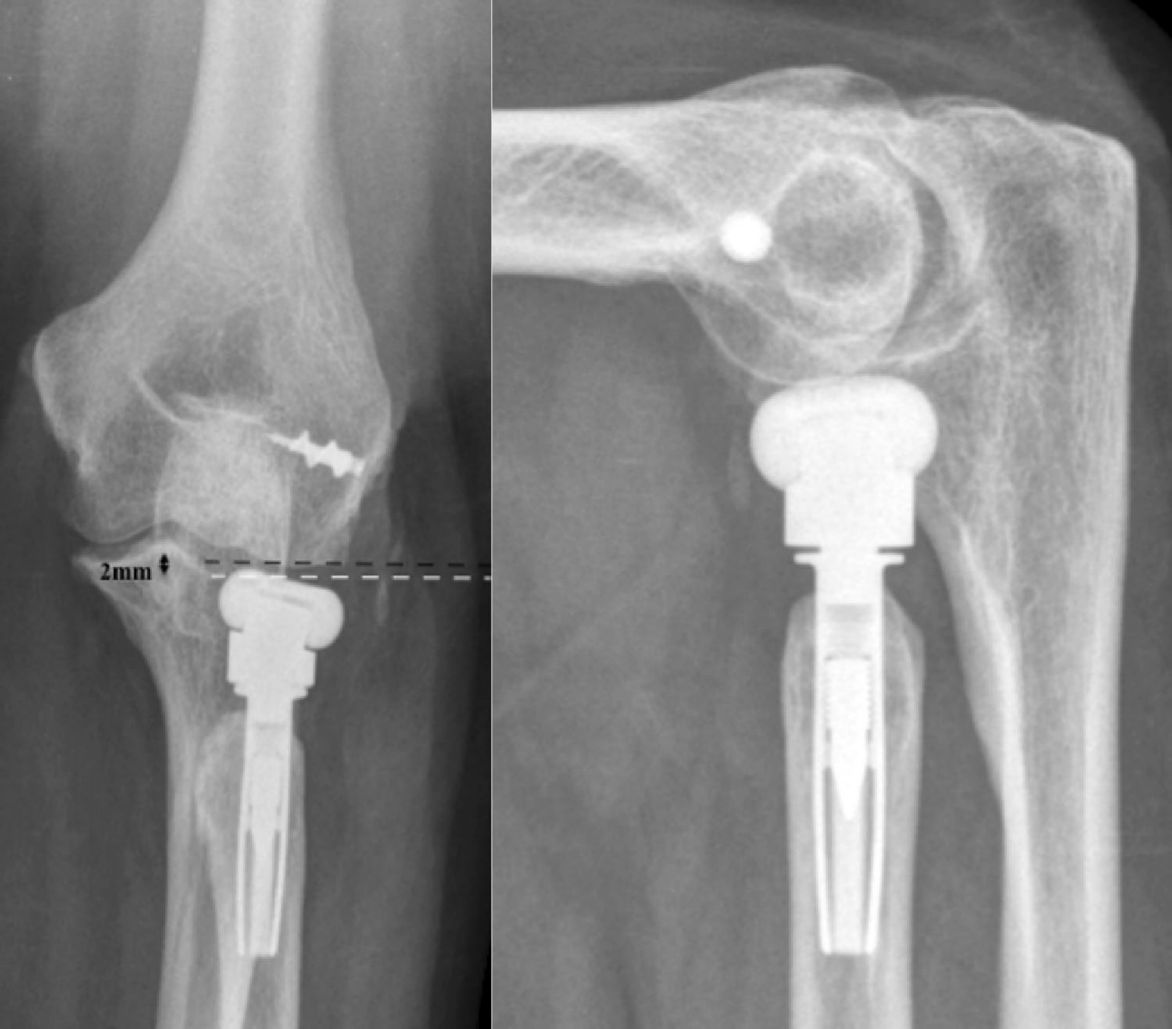
Type I A: Overlengthening of 2 mm (black dotted line), the bony structure of the capitulum is intact

**Fig. 7:**
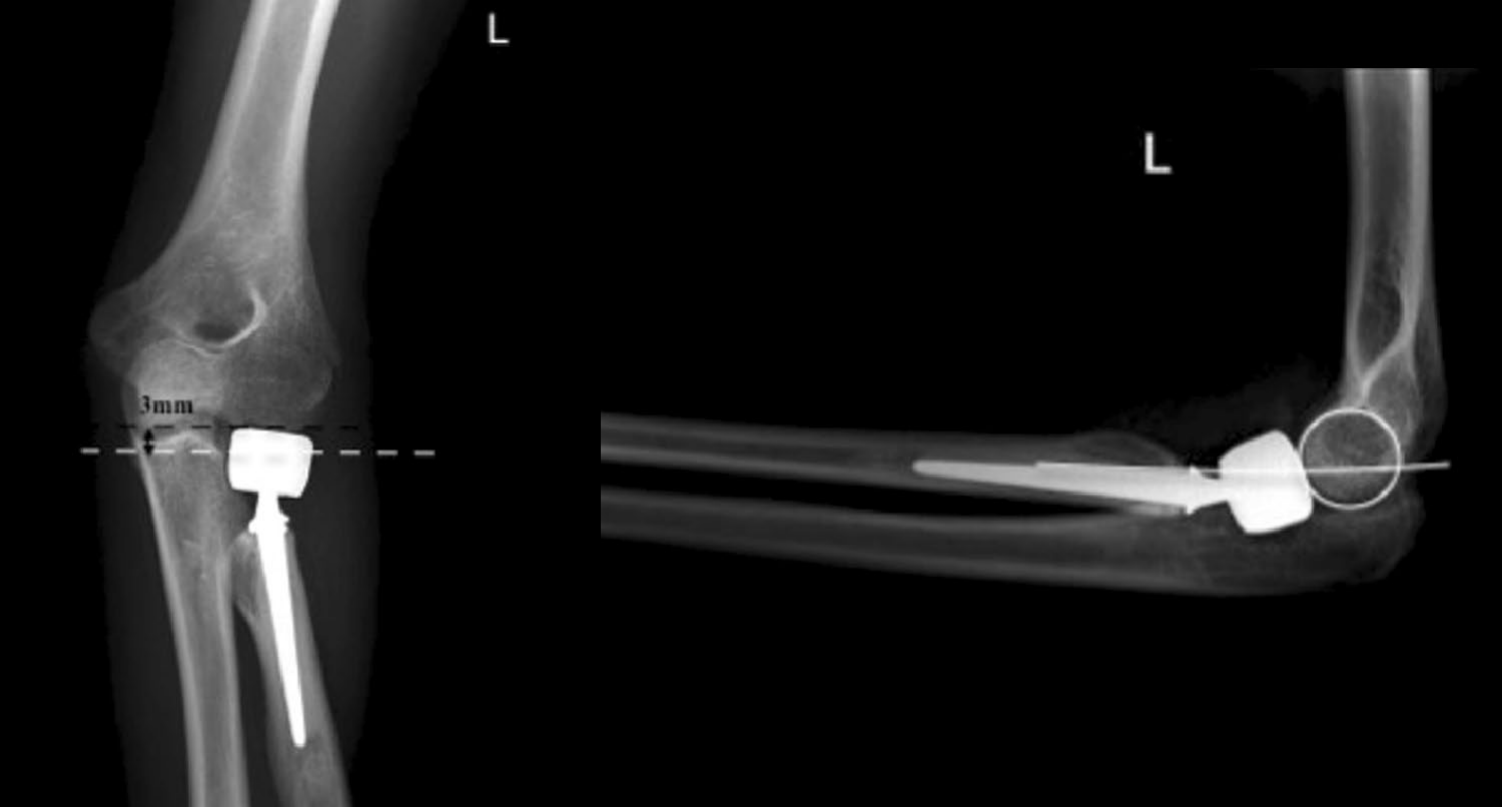
Overlengthening Type II A, with the radial head implant overreaching the lateral coronoid (white dotted line), with stable articulation. The subchondral bone of the capitulum is intact

The classification is based on the native anatomical relation of the radial head to the lateral edge of the coronoid. Therefore, direct measurement of that relation is necessary, identifying whether the radial head prosthesis overreaches the lateral edge of the coronoid and to quantify it in millimetres. To achieve this measurement, imaging with an orthogonal view on the PRUJ is needed. In the case of X-ray imaging, extension deficit, which is often present in patients after radial head prosthesis surgery may obscure adequate imaging. Therefore, we recommend inclining the X-ray tube according to the extension deficit, to bring the central-beam of the X-ray tube in an orthogonal direction onto the forearm and not onto the distal humerus. This will result in an a.p. view on the PRUJ. In CT-imaging, 3D-reconstructions help finding the adequate plane independent of elbow position. At hand of such an orthogonal view, the height difference of the radial head prosthesis surface and of the lateral edge of the coronoid is quantified (Figs. 5, 6, 7).

**Table:** 

Type	Description	Subtype	
		A	B
I	Overlengthening of up to 2 mm	Without erosion of the subchondral bone of the capitulum	With erosion of the subchondral bone of the capitulum
II	Overlengthening of more than 2 mm, with stable articulation		
III	Overlengthening with subluxation or dislocation of the elbow		
IV	Dynamic overlengthening due to longitudinal instability of the forearm		

Type I (A) and (B)

Overlengthening of up to 2 mm without (A) or with (B) signs of erosion of the capitellar cartilage in imaging studies (Fig. 6).

Type II (A) and (B)

Overlengthening of more than 2 mm, with a stable joint, without (A) or with (B) signs of erosion of the capitulum in imaging studies (Fig. 7).

Type III (A) and (B)

**Fig. 8:**
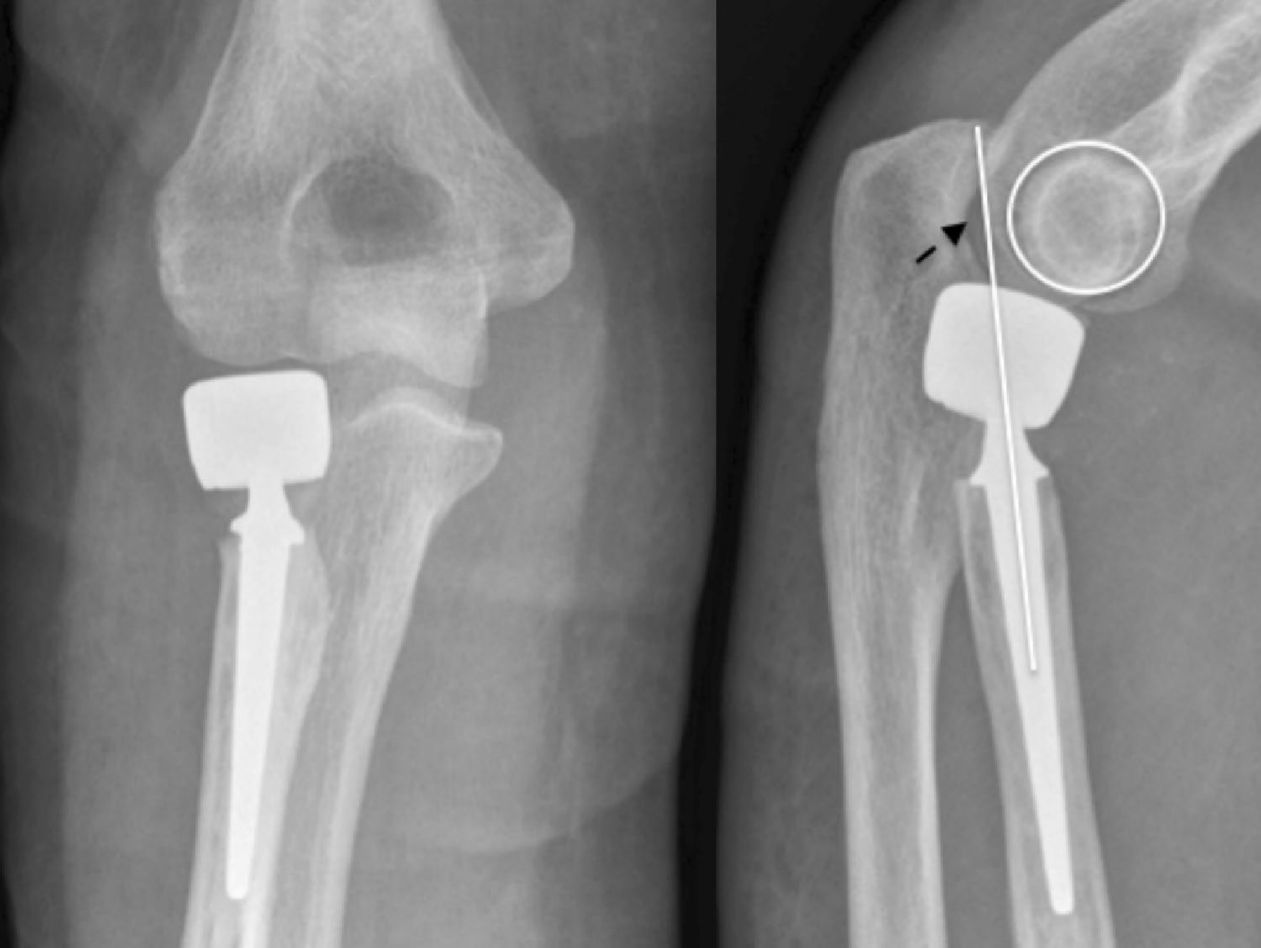
Overlengthening Type III A in a bi-polar radial head prosthesis, with a positive drop-sign, indicative of subluxation of the joint. The subchondral bone of the capitulum is intact

Overlengthening with subluxation or dislocation of the elbow, without (A) or with (B) erosion of the subchondral bone of the capitulum (Fig. 8).

Type IV (A) and (B)

**Fig. 9:**
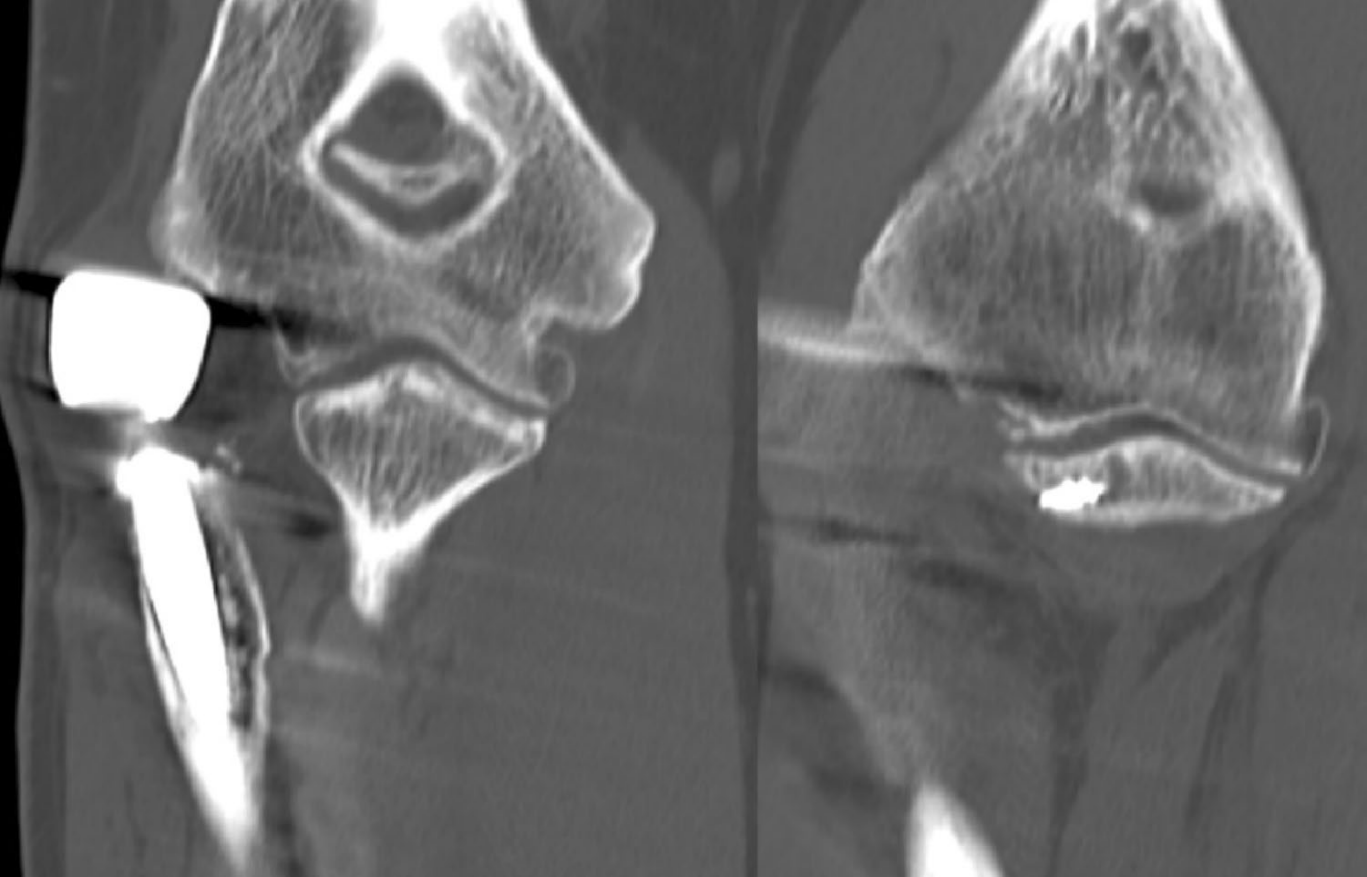
Type IV: dynamic overlengthening due to longitudinal instability of the forearm in a chronic Essex-Lopresti-lesion. A bi-polar radial head implant had been implanted in this patient. Due to overlengthening, the implant had grinded into the capitulum, resulting in substantial bone loss

Dynamic overlengthening due to longitudinal instability of the forearm, without (A) or with (B) erosion of the subchondral bone of the capitulum (Fig. 9).

### Treatment strategies

**Fig. 10:**
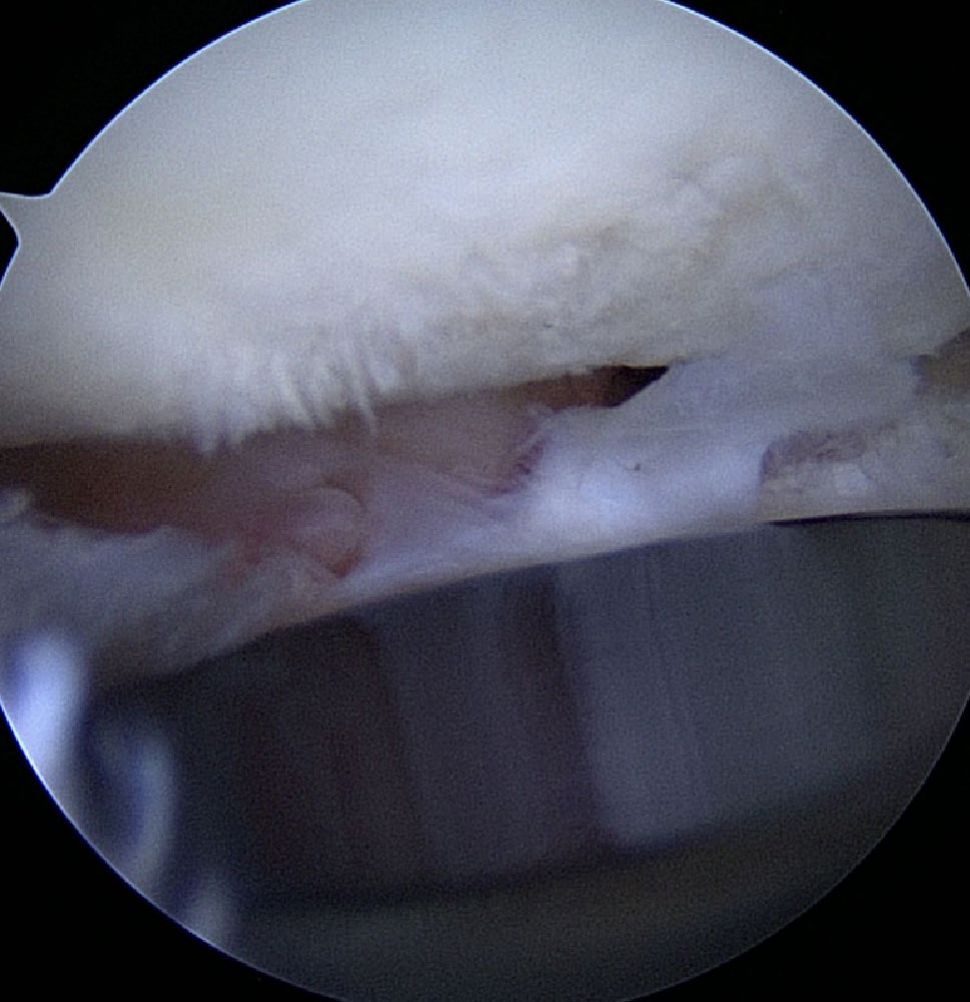
Arthroscopy in overlengthening Type I with a Mopyc Prosthesis (Wright Medical/Tornier), with the capitulum showing minor cartilage degeneration

**Fig. 11:**
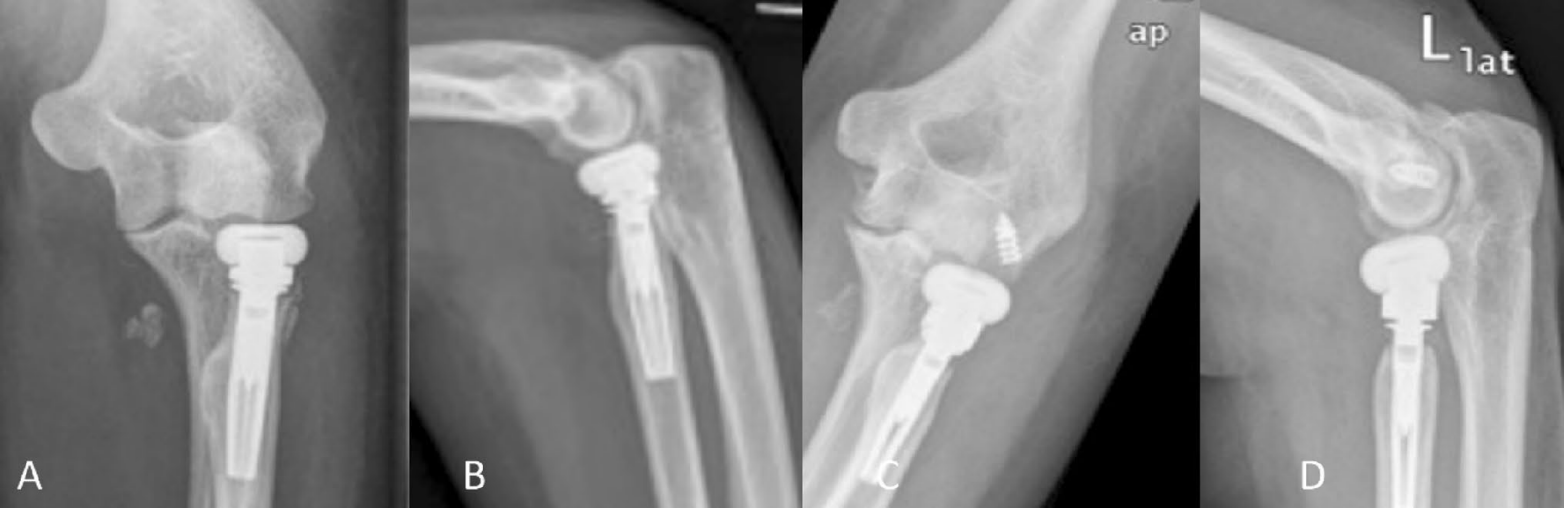
Overlenghtening IA, with moderate erosion of the capitulum (Arthroscopy findings s. Figure 10), where revision arthroplasty was elected, with augmentation of the lateral ligamentous complex

Due to the significant changes to joint mechanics and the erosive effects on capitellar cartilage, overlengthening of radial head prostheses often is subject to revision elbow surgery. Several surgical options from revision radial head prosthesis to resection- or interposition arthroplasty are available. As several factors influence the choice of revision technique, we adhere to the presented classification system. Concerning the lower grades of overlengthening, so far it is not clear whether an amount of < 2 mm is of clinical relevance. As we regularly see patients with low-grade overlengthening of up to 2 mm with significant clinical problems, we assort such cases to group I. We believe it is important to acknowledge also low-grade overlengthening as a pathology. We treat patients with Type I overlengthening initially conservatively. The patients are brought to physiotherapy and manual therapy, paired with a trial period of load relief for 6 weeks. If the patient´s pain does not respond to conservative therapy or if stiffness necessitates revision surgery, we perform arthroscopy with detailed analysis of joint mechanics and evaluation of the osteo-cartilaginous condition of the capitulum. If in such cases overlengthening is identified as causative for the clinical symptoms and if the capitulum does not show significant bony erosion (Type IA) (Fig. 10), we see revision radial head prosthesis, levelled at the lateral edge of the coronoid as an appropriate option (Fig. 11).

If erosion of the subchondral bone of the capitulum is found (Type IB) it is an option to remove the prosthetic component and check for valgus stability and for longitudinal stability. If the joint is stable in these regards, one can elect not to re-implant a prosthesis. By that the capitulum is unloaded, and a potential cause for pain and ongoing inflammation is taken from the joint. Another option is to intentionally “underlengthen” the implant. The idea is based on a biomechanical study we performed, to evaluate a beneficial effect of unloading the radio-humeral joint in the case of arthriti [28]. Shortening of the proximal radius by 3 mm leads to a significant unloading of the radio-humeral joint without affecting valgus stability. Maybe, unloading of a radial head prosthesis by deliberately placing it below the lateral edge of the coronoid can help reducing symptoms in patients with erosion of the capitulum. However, if we aim for under-lengthening of a radial head prosthesis, we do not take away more than 2 mm, as further lowering of the implant may lead to conflict with the lower edge of the lesser sigmoid notch. Moreover, the patient has to be informed pre-operatively in a written consent, about the clinical consequences of resection or shortening of the proximal radius. These consequences mainly are the development of tardy valgus- and longitudinal instability, resulting for example in medial elbow pain or wrist pain. If after removal of the radial head implant the elbow is unstable, ligament reconstruction or augmentation is mandatory.

**Fig. 12:**
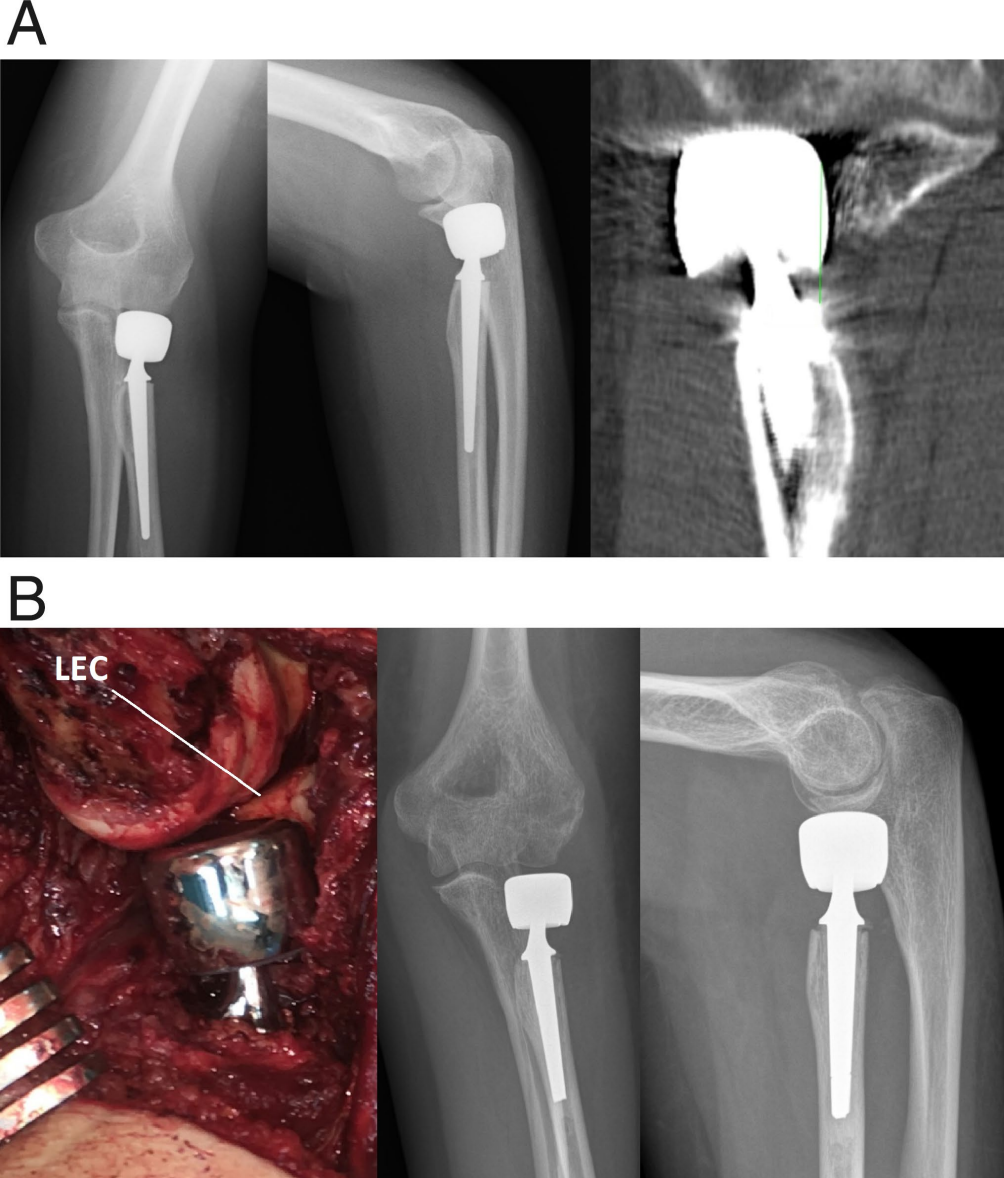
**a** Case of overlengthening IIB. **b** After revision with again a cemented bi-polar implant, which was implanted slightly below the lateral edge of the coronoid (LEC), resulting in a gap between radial head component and capitulum

If overlengthening of more than 2 mm is present (Type II A or B) (Fig. 12a, b), we regularly perform revision surgery. In these cases, often the capitulum is already eroded by the increased contact pressure, leading to ongoing inflammation with its typical clinical consequences. Conservative therapy in our experience does not lead to improvement in the case of significant overlengthening. As the capitulum is often already eroded, revision strategies must be evaluated carefully. Implantation of a further radial head prosthesis potentially ends up in recurrence of the symptoms, due to repetitive contact between the prosthesis and the eroded capitulum [29]. Hence, an attractive revision option again can be removal of the prosthesis, while respecting the aforementioned pre-conditions to do so. If sole removal is not an option, one can choose interposition arthroplasty to achieve an abutment between the radial stump and the capitulum. Interposing the anconeus muscle is a viable option, as shown by Rahmi et al. [29]. A limiting factor may be scarring of the anconeus due to previous surgeries, rendering the muscle inadequate to be mobilised and interposed. Also, if due to bony resection or bone-loss the gap between capitulum and the radial stump is substantial, a filling effect can not be achieved with the anconeus. A further option of treatment would be prosthetic replacement of both the radial head and the capitulum [30, 31]. With replacement of an eroded capitulum, the potential pain generator is removed. However, radio-capitellar arthroplasty is not without risks and has been shown to come along with severe complications [32].

In overlengthening of Type III, additionally ligamentous deficiencies are regularly present and should be addressed. As overlengthening in these cases is severe, and the joint is subluxed or even dislocated by the increased contact pressure, erosion of the capitulum is usually always present. Even severe abrasion with significant bone loss can be seen. Surgical revision is warranted in these cases. The first step is removal of the prosthesis. Following that, stability of the joint without the irritating prosthesis should be assessed thoroughly. In overlengthening of Type III, subluxation of the joint happens due to a severely increased length of the proximal radius. By that, the joint is distended and the medial and lateral ligamentous complex is tensioned [22]. Therefore, it is often necessary to address the medial and lateral ligaments. As the cases usually do not present acutely, we prefer to perform ligament reconstruction with autologous tendon grafts, augmented with internal bracing to secure the construct. Patients who do not have sufficient musculature to support joint stability, e.g., adipose arms, we consider additional stabilization with an external fixator for at least 6 weeks after surgery, to protect the repair.

**Fig. 13:**
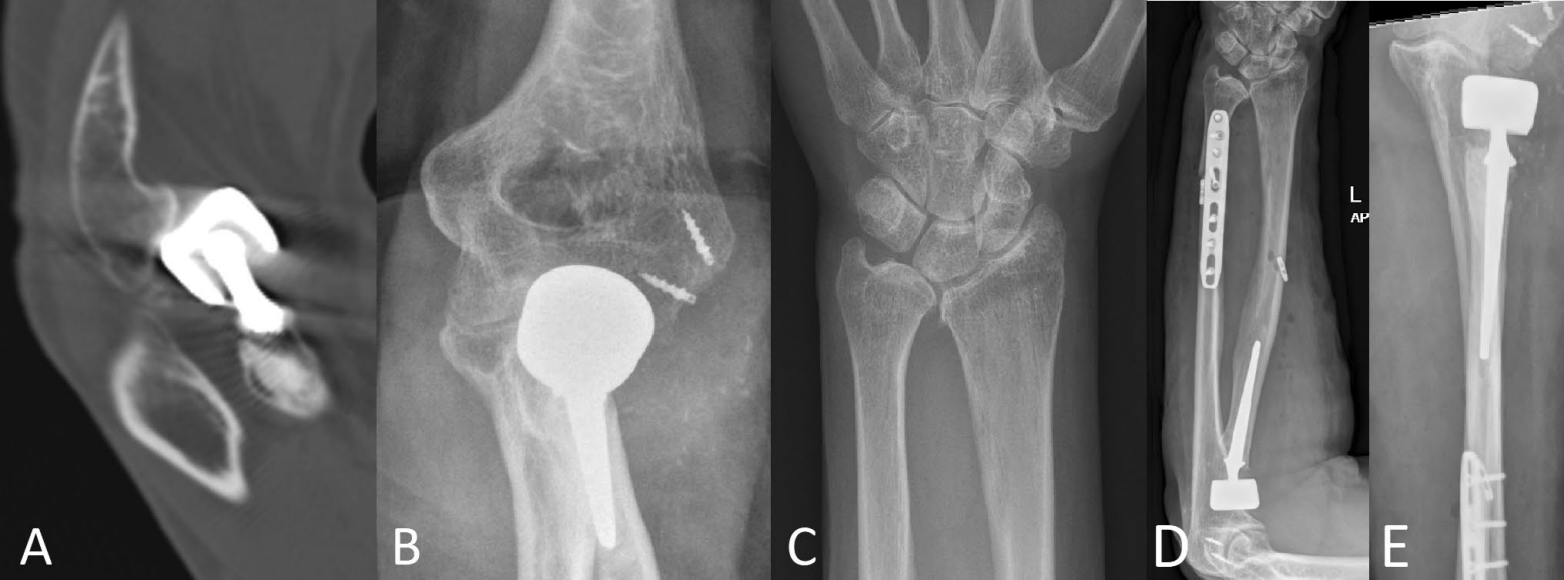
Case of dynamic overlengthening (Type IV). The patient had been treated for an Essex-Lopresti lesion with a bi-polar implant. Due to the steady proximal push, the implant had grinded into the capitulum and lead to an erosion (**a**, **b**). The wrist showed a typical ulnar positive variant (**c**). Due to the complex lesion, lowering of the prosthesis, ulnar shortening osteotomy and reconstruction of the central band of the interosseous membrane had to be performed (**d**, **e**)

**Fig. 14:**
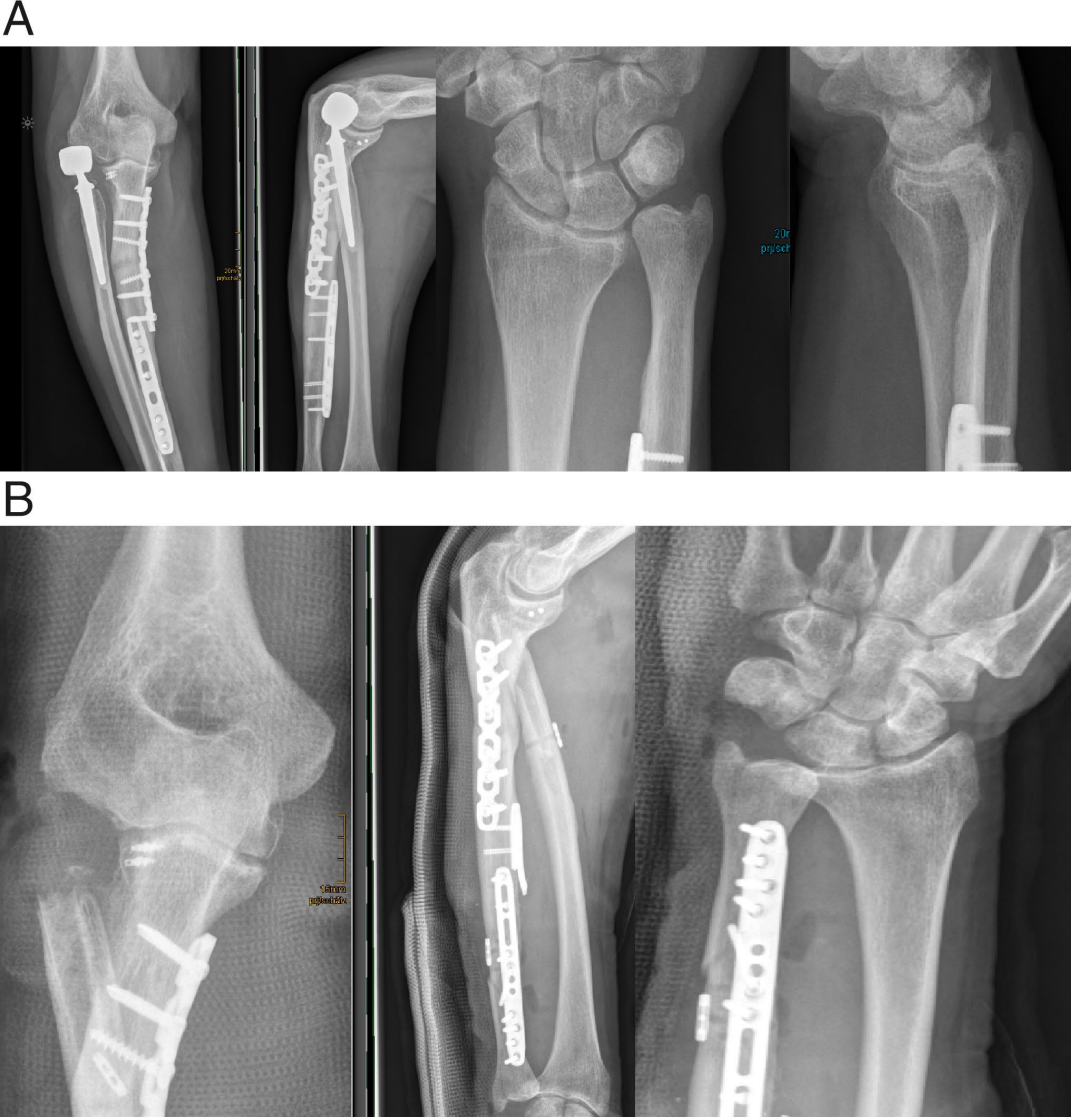
Another case of dynamic over-lengthening, the 44-year-old male patient presented with crepitus at the level of the implant, and pain at the elbow and wrist, after previous surgeries. Imaging showed erosion of the capitulum due to massive dynamic overlengthening, and positive ulnar variance. **a** The patient was treated with implant removal, ulnar shortening and reconstruction of the central band of the IOM (**b**)

In overlengthening of Type IV, the underlying pathology has to be addressed. As longitudinal instability of the forearm would repetitively lead to increased contact pressure between the prosthesis and the capitulum, relieve of symptoms cannot be achieved by lowering the present implant alone. Type IV usually comes with severe abrasion of the capitulum. To stabilize the proximal push of the radius, we reconstruct the central band of the interosseous membrane. Several surgical techniques have been presented to address the issue of longitudinal instability of the forearm [33, 34]. Therefore, autologous tendons or artificial fiber systems are available. If positive ulnar variance is present, ulnar shortening has to be considered accordingly (Fig. 13). In cases with longitudinal instability, the capitulum often shows severe abrasion or bone loss, due to the high contact pressures. In such cases, revision radial head prosthesis is not recommended and removal of the implant with longitudinal stabilization and shortening of the ulna is a salvage option (Fig. 14).

## Discussion

Correct sizing and lengthening of prosthetic components is a known primary technical goal in any musculoskeletal replacement surgery. Oversizing, Overstuffing and Overlengthening are well-identified complications in Knee, Hip and Shoulder arthroplasty [35, 36]. The radio-humeral joint is one of intricate anatomy and biomechanics, too. Several clinical and biomechanical studies have reported the problem of overlengthening in radial head arthroplasty and have given strategies for identification and treatment. Van Glabbeek et al. have found significant alterations of elbow biomechanics with overlengthening of the radial column in six human cadaveric specimens [6]. The authors concluded that radial head prostheses would need to be implanted with high accuracy and reproducibility. In a further biomechanical study, van Glabbeek et al. investigated the effect of overlengthening of a radial head prosthesis on the rotational position of the ulna, compared to the native state [7]. The group showed that overlengthening of more than 2.5 mm led to varus malposition of the ulna, together with external rotation. Both reports clearly recommend implantation of radial head prostheses at the correct height. Concerning the radial head, revision of painful overlengthening to a total elbow prosthesis is not a favourable option, as total elbow arthroplasty shows high complication rates and limited survival time in younger trauma patients, which are typically affected by overlenghtening [37–39]. As we stated, clinical series on overlengthening of radial head prostheses are rare and no reliable standards for the treatment are available. The presented descriptive classification system aims to raise the awareness of the clinical entity of radial head prosthesis overlengthening. The paper has several limitations, as the presented classification has not yet been evaluated at hand of a clinical series. Hence, it is not clear, except from very little literature, whether resection of radial head prostheses for painful overlengthening alone or combined with interposition does offer good long-term results. Nor it is clear, whether radio-capitellar arthroplasty shows a reasonable rate of complications and favourable long-term outcomes, to solve radial overlengthening with painful capitellar erosion. Also, it is not known whether deliberately reducing the length of the radial head implant below the recommended level of the lateral edge of the coronoid may reduce symptoms of capitellar erosion by a radial head implant. Our biomechanical study on the shortening of the radial head in case of symptomatic radio-humeral arthritis supports at least the idea. [28] It showed no negative biomechanical effects of gentle shortening of the proximal radius regarding stability, but it did reduce the contact pressure. Accordingly, the treatment recommendations given in the present paper are based only on the experiences of our clinical practice. Hence, it is of importance to further shed light into the clinical entity and to improve the knowledge concerning the topic. To the best of our knowledge, this is the first review describing a classification system on overlengthening of radial head prostheses.
